# Identification of Alpha and Beta Coronavirus in Wildlife Species in France: Bats, Rodents, Rabbits, and Hedgehogs

**DOI:** 10.3390/v9120364

**Published:** 2017-11-29

**Authors:** Elodie Monchatre-Leroy, Franck Boué, Jean-Marc Boucher, Camille Renault, François Moutou, Meriadeg Ar Gouilh, Gérald Umhang

**Affiliations:** 1 ANSES, Laboratoire de la rage et de la faune sauvage, 54220 Nancy, France; franck.boue@anses.fr (F.B.); jean-marc.boucher@anses.fr (J.-M.B.); camille.renault20@gmail.com (C.R.); gerald.umhang@anses.fr (G.U.); 2 ANSES, ENVA, 94701 Maisons-Alfort, France; francoismoutou@orange.fr; 3 Université de Normandie, EA 2656, GRAM—Groupe de Recherche sur l’Adaptation Microbienne, UNICAEN/UNIROUEN, 14000 Caen, France; meriadeg.legouil@normandie-univ.fr; 4 Institut Pasteur, Infection et Epidemiologie, Unité Environnement et Risques Infectieux, 75015 Paris, France

**Keywords:** coronavirus, wildlife, wild rabbits, hedgehogs, bats, rodents, France, genetic diversity

## Abstract

Coronaviruses are closely monitored in the context of emerging diseases and, as illustrated with Severe Acute Respiratory Syndrome coronavirus (SARS-CoV) and Middle East Respiratory Syndrome-coronavirus (MERS-CoV), are known to cross the species barrier and eventually to move from wildlife to humans. Knowledge of the diversity of coronaviruses in wildlife is therefore essential to better understand and prevent emergence events. This study explored the presence of coronaviruses in four wild mammal orders in France: Bats, rodents, lagomorphs, and hedgehogs. *Betacoronavirus* and *Alphacoronavirus* genera were identified. The results obtained suggest the circulation of potentially evolving virus strains, with the potential to cross the species barrier.

## 1. Introduction

Coronaviruses (CoVs) infect a wide variety of animals and are common throughout the world. They cause respiratory, enteric, hepatic, and neurological diseases with variable severity, from asymptomatic to severe. Coronaviruses that infect mammals (except pigs) belong mainly to two genetic and serologic groups: The *Alpha*- and *Betacoronavirus* genera.

Along with *Paramyxoviridae* and the influenza viruses, the *Coronaviridae* family is one of the three viral families closely monitored in the context of emerging diseases. With Severe Acute Respiratory Syndrome coronavirus (SARS-CoV) and Middle East Respiratory Syndrome (MERS-CoV), coronaviruses have shown their ability to move from wildlife to domestic animals or to humans. In this context, describing the coronaviruses circulating in wildlife in a specific area is the first step to being readily responsive in the event of emergence. However, data concerning wildlife species harboring coronaviruses in Europe and especially France are currently very limited. Bat coronaviruses have been studied because of the SARS-CoV and MERS-CoV epidemics. In Europe, both beta-CoVs and alpha-CoVs have been found in bats [1,2]. To our knowledge, only four bat species among the 35 species living in France have been screened for *Coronaviridae* [2].

Since all these novel alpha- and beta-CoVs were found in insectivorous bats, the search in Western European hedgehogs (*Erinaceus europaeus*), as an insectivorous mammal belonging to a related order of Chiroptera, resulted in the identification of a novel *Betacoronavirus*, Eri-CoV, detected from animals raised in an animal shelter in northern Germany [3]. Other wildlife species appear to be relevant to identify the potential presence of *Coronavirinae* given, for instance, the recent description in wild rodents of infection by *Alphacoronavirus* [4]. Another species of interest is wild rabbits (*Oryctolagus cuniculus*) because of beta-CoV identification in domestic rabbits in China [5], which have wild European rabbits as ancestors.

The aim of this study was to explore the presence of *Coronaviridae* in specific relevant species of wildlife in France, in order to produce an overview of the reservoir of potential emerging *Coronaviridae* from wildlife and of the species that host them.

## 2. Materials and Methods

### 2.1. Sample Collection

**Figure 1:**
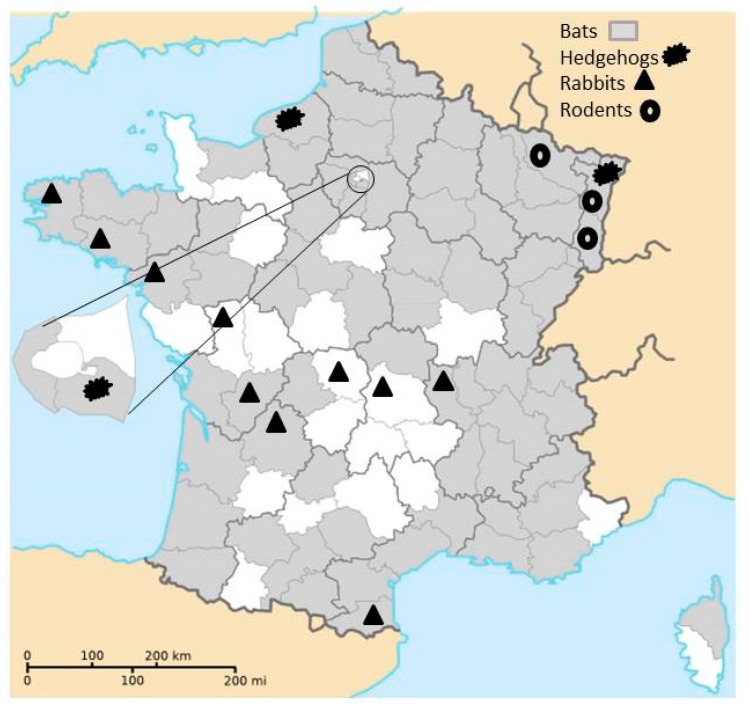
Different types of collected samples by French administrative department (excluding French Guyana in South America).

Samples of intestinal content were used for the detection of CoVs in bats, rodents, and rabbits. Regarding studies about the detection of CoVs, intestinal samples were tissues with the highest CoV RNA concentration in hedgehogs [3] and were successfully used to detect CoV in bats [6] and rabbits [5]. Intestinal samples were collected from carcasses of these animals as follows:

- From 2013 to 2015, intestinal samples from carcasses of bats were collected within the lyssaviruses surveillance in France and originated from 74 of the 101 French administrative department (with only one overseas French department concerned, French Guyana, Figure 1).
- From 2014 to 2016, rodents were trapped in five areas of eastern France (Figure 1) in the context of other studies. Rodents were captured using pieces of carrot and sunflower seeds as bait in different kinds of traps adapted to species. Captured animals were euthanized in accordance with the French Animal Protection Law and Directive 2010/63/EU of the European Parliament and of the Council on the protection of animals used for scientific purposes (identification code of the approval (29 September 2016) project AP AFIS#2939-20160106142231, name of the ethics committee: Cometh).
- From 2007 to 2009, intestinal samples from rabbits (*Oryctolagus cuniculus*) collected in 10 departments by the French National Hunting and Wildlife Agency for another research project were analyzed (Figure 1). Intestinal samples were taken from animals killed by hunters during rabbit hunting. Therefore, no wild animal was killed specifically for the purpose of this study.

Intestinal samples were stored at −20 °C without additives.

Samples of hedgehogs (*E. europaeus*) were fecal samples collected in three animal shelters in 2015 and 2016 (Figure 1). Hedgehog droppings were sampled on the first or second day after their arrival at the animal shelter. Fecal samples were stored at −20 °C in UTM-RT™ (Universal Transport Medium, Copan diagnostics, Murrieta, CA, USA).

### 2.2. RNA Extraction and RT-PCR of the Partial RNA-Dependent RNA Polymerase Protein (RdRp) Gene

A Nucleospin Viral RNA isolation kit (Macherey-Nagel, Hoerdt, France) was used for RNA extraction from stools or from intestinal homogenate. Reverse transcription was performed from 8 µL of RNA with RT Superscript III and random hexamer primers. Amplification by PCR was carried out as described previously [7], by using a semi-nested PCR targeting the RdRp conserved coding region using 5 µL of cDNA in a final volume of 50 µL. PCR products were sent for sequencing by private companies (GenoScreen and Genewizz, Lille, France). Nucleic sequences of 125 bases corresponding to the RdRp gene were recovered from all positive samples.

### 2.3. Phylogenetic Analysis

**Figure 2:**
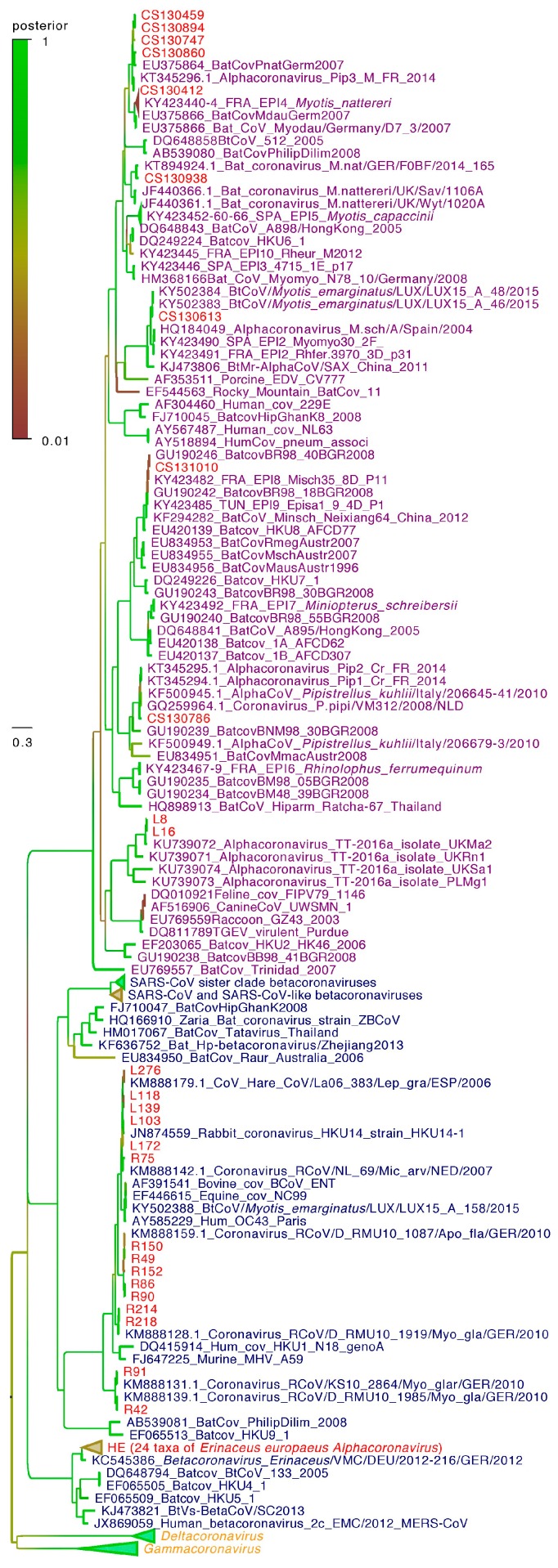
Bayesian phylogeny of 50 genetic sequences of viruses detected in the study with 127 representative sequences of the whole diversity of the group of *Coronavirinae*. Selected coronavirus RdRp sequences, including some extracted from full genomes, were aligned with original sequences from this study in SEAVIEW v4.6. [8]. Statistical support (posterior probability) of nodes are depicted using a gradual color code of the tree, with green indicating significant posterior probability values (>0.95). Genbank identification numbers, strain names, and the main information are written in taxa labels. Taxa labels of *Alphacoronavirus*, *Betacoronavirus* and of the clade grouping *Gammacoronavirus* and *Deltacoronavirus* are in purple, blue, and orange, respectively. Viruses detected in this study are depicted in red (HE: Sequences from hedgehogs, R: Sequences from rodents, CS: Sequences from bats; and L: Sequences from rabbits).

The CoV sequences found in this study were deposited in the DNA Data Bank of Japan (DDBJ) under accession numbersLC334347 to LC334385 and LC334392 to LC334408. Sequence similarity to known sequences was determined using Basic Local Alignment Search Tool (BLAST) nucleotide analysis against the GenBank database. The phylogenetic tree (Figure 2) was inferred using sequences obtained from positive samples aligned with coronavirus sequences representing the whole diversity of the group. Sequence multi-alignments were performed in SEAVIEW v4.6 [8]. Phylogenetic reconstruction using maximum likelihood was done using PhyML v3.0 [9]. The GTR+G+I (General Time Reversible) substitution model was selected as the optimal model by a function implemented in MEGA7 [10] and a discrete gamma distribution with four categories was used for rate heterogeneity. Trees were also inferred using the Bayesian approach implemented in the Beast package, with both GTR (General Time Reversible) and TN93 (Tamura-Neï 1993) optimum models, gamma distribution, and invariable sites. The coalescent constant size model was used and the clock parameters were set to the uncorrelated lognormal relaxed clock. The length of the chain was then set to 30 million iterations in order to produce ESS (Effective Sample Size) superior to 200. The maximum credibility tree with the branch length in number of substitutions was defined from ten thousand trees after a discard of 10% and was edited using FigTree (version 1.4.3 on http://tree.bio.ed.ac.uk/software/figtree/).

### 2.4. Statistical Analysis

Confidence intervals for seroprevalence were estimated using the Wilson method [11] in the Epitools calculator (http://epitools.ausvet.com.au/).

## 3. Results

### 3.1. Bats

Intestinal samples from 504 bats were analyzed (Table 1). Twelve bats were found to be infected by a coronavirus. Five sequences from *Pipistrellus pipistrellus* and two from *Pipistrellus* sp. had a 94% to 99% nucleic acid sequence identity with alpha-CoVs of *Pipistrellus* from France and Germany (GenBank KT345295, KT345296 and EU375868). Two *Myotis emarginatus* were found to be infected by coronaviruses with an identity of 100% with sequences of alpha-CoVs found in *Myotis emarginatus* in Luxembourg (GenBank KY502401). The coronavirus found in one *Myotis nattereri* exhibited a 95% identity with an unclassified coronavirus found in *Myotis nattereri* in Hungary (GenBank KJ652333). Concerning *Miniopterus schreibersii*, the nucleic sequences of the two infected animals had an identity of 99% and 98% with coronaviruses found in the same species in Bulgaria (GenBank GU190247 and GU190241).

### 3.2. Hedgehogs

Fecal samples from 74 hedgehogs were analyzed and 37 samples were positive. The estimated prevalence for all the animal shelters was 50% (CI95%: 38.89; 61.11), with no statistical difference between the three animal shelters.

All sequences were strongly homogeneous with a maximum of four differences at the nucleotide level. The nucleic sequences detected from hedgehogs had an identity from 96% to 98% with the sequence of coronavirus (KC545383) found in the hedgehogs in Germany.

### 3.3. Rodents

Of the 330 trapped rodents (Table 2), most of the animals belonged to *Apodemus* spp. (*n* = 206) and *Myodes glareolus* (*n* = 80), and were collected at four sites (Bitche, Forbach, La-Petite-Pierre, and Murbach) in mixed coniferous and deciduous forests. Other species of rodents were *Arvicola terrestris* (*n* = 35) and *Microtus* spp. (*n* = 9), and were mainly collected in an apple orchard in Wissembourg (Bas-Rhin).

Twenty-one samples were positive, represented by five *Myodes glareolus* (6.25% (CI 95%: 2.70; 13.81)) and 16 *Apodemus* spp. (7.77% (CI 95%: 4.84; 12.24)). Positive samples were found each year and in each area (Table 2), except in Wissembourg. All the sequences were very close (identities from 94% to 98%) to five sequences of unclassified coronavirus detected in 2010 in Germany in *Myodes glareolus* and *Apodemus* spp. (KM888128, KM888131, KM888139, and KM888159), and in 2007 in the Netherlands in *Microtus arvalis* (KM888142). Aligned sequences were separated into two groups. Fifteen sequences with a maximum difference of two nucleotides formed the first group detected from *Apodemus* spp. samples. The other six sequences of the second group with 27 to 33 nucleotides different from the R152 sequence of the first group were detected from samples of *Myodes glareolus*, except one in *Apodemus* spp.

### 3.4. Rabbits

A total of 291 intestinal samples from rabbits were analyzed. Twenty-two animals were found to be infected, corresponding to a global estimated prevalence of 7.56% (CI 95%: 5.05; 11.18), which differed from one department to another from 0% (CI 95%: 0.00; 29.91) to 16.66% (CI 95%: 8.32; 30.60).

Seventeen sequences were very close (with identities from 94% to 98%) to the beta-CoV RbCoV HKU14 (5) and to an alpha-CoV identified in hares (*Lepus* sp.) and rabbits in Spain (with identities from 97% to 98%) (GenBank Nos. KM888169 to KM888181). Five sequences were very close (with identities from 96% to 97%) to the alpha-CoV identified by Tsoleridis et al. [4] in rodents (KU739072). Both beta- and alpha-CoVs were identified in rabbits from the same geographical area and collected the same year. For each genus, nucleic sequences showed strong homogeneity, with a maximum of five differences at the nucleotide level.

### 3.5. Phylogenetic Diversity

Phylogenetic analyses (Figure 2) of the partial RdRp gene revealed that all coronavirus sequences from bats belonged to the *Alphacoronavirus* genus. Sequences of alpha-CoVs detected in France from *Miniopterus schreibersii* (CS131010) clustered with sequences of alpha-CoVs detected in the same species in Germany. Likewise, sequences of alpha-CoVs from *Myotis emarginatus* from France (CS130613) clustered with sequences of alpha-CoVs detected in the same species in Luxembourg. Similarly, sequences of alpha-CoVs from *Myotis nattereri* in France (CS130938) clustered with sequences from the same species in Germany and England. Sequences of alpha-CoVs from *Pipistrellus* sp. in France (CS130412, CS130747, CS130894, CS130459, CS130860, and CS130786) were found in two distinct groups, well supported by Likelihood Ratio Test analysis and posterior probabilities. The other sequences of European alpha-CoVs from *Pipistrellus* sp. (KT345294, KT345295, and KT345296 in France, KF500945 in Italy, and GQ259964 in the Netherlands) also clustered into these two distinct groups.

Nucleic sequences from hedgehogs (HE on Figure 2) were highly homogeneous and clustered with one of the other sequences of beta-CoV detected from hedgehogs in Germany. The sublineage of hedgehog sequences, human MERS-CoV, camel MERS-CoV-like, and bat MERS CoV-like sister clade is different from the other beta-CoVs, as shown by its well-supported Likelihood Ratio Test analysis and posterior probabilities.

All sequences of beta-CoV detected from rodents in France were in the same group of sequences as beta-CoV from wild rabbits detected in the same country (L103, L118, L139, L172, L276) and domestic rabbits in China (GenBank No. JN874559). Sequences of beta-CoV from *Apodemus* sp. in France (R49, R86, R90, R150, R152) were in the same subgroup, whereas sequences from *Myodes glareolus* were found in three distinct subgroups: Two (R42, R91, R214, R218) with sequences of beta-CoV from *Myodes glareolus* from Germany (KM888128, KM888131 and KM888139); and the third with a sequence (R75) from *Microtus arvalis* from the Netherlands (KM888142).

The two sequences of alpha-CoV from rabbits from France (L8, L16) were in the same group of alpha-CoVs from rodents in the United Kingdom (GenBank Nos. KU739072, KU739073, KU739074, and KU739071).

## 4. Discussion

This study described the presence of coronavirus infection in four wild mammal orders in France. Our results suggest that coronaviruses seem to be relatively common in these species, regardless of the place and time of sampling. The sequence length in our study was short because samples originated from other works for other purposes (except hedgehogs); however, interesting conclusions can be suggested. Studies about coronaviruses in bats are numerous in comparison with other wild mammals. In Europe, alpha-CoVs have been found in *Pipistrellus* sp. (France, Hungary, Romania, Germany, Spain, Italy, and the Netherlands), *Myotis* sp. (Hungary, Germany, Spain, the United Kingdom, and the Netherlands), *Rhinolophus* sp. (Bulgaria and Hungary), and *Nyctalus* sp. (Spain and Bulgaria) [2]. In this study, seven *Pipistrellus* (six *P*. *pipistrellus* and one *Pipistrellus* sp.) were found to be positive in several geographically distant parts of the country (East, West and North of France). Two *Myotis emarginatus* in the same area in the East of France, one *Myotis nattereri* in the North of France, and two *Miniopterus schreibersii* were found in two areas in the South and East of the country. The number of positive samples by site (except for *Miniopterus* with two positive samples out of five) was highly dependent on the number of collected samples by site. The collection concerned 23 species of bats, out of the 35 known in mainland France. The collection of dead bats through the French network for lyssaviruses surveillance is performed by volunteers with no minimum sample sizes in each department, explaining the heterogeneous distribution of samples and the respective abundance of populations in biotopes close to humans. The use of this network allowed for better distribution of the dead animal samples across the national territory, compared to colony by colony collection. Nevertheless, the detection of the RNA sequence of RdRp in the infected animals was highly dependent on the state of the carcasses when discovered, and on storage before being dispatched to the laboratory.

The overall prevalence in bats of 2.4% (CI 95%: 1.37; 4.12) was consistent with other studies in European countries, which have reported figures from 0% ([12] in Belgium) to 17.1% ([13] in the UK).

This was the second report of *Myotis emarginatus* infection by coronavirus inside the usual range of this bat; the first was in Luxembourg [14]. Interestingly, both cases reported here came from a department very close to Luxembourg. This species was rarely (about 20 samples) concerned in previous studies [15,16,17,18], but it would be relevant to study coronaviruses in these bats from other parts of its range to confirm the existence of a novel RdRp-grouping unit (RGU), as suggested in a recent study [14]. In addition, our new nucleic sequences of coronavirus identified from *Myotis emarginatus* belong to *Alphacoronavirus*, like all sequences previously detected from other *Myotis* bats in European countries [13,16,17,18,19,20,21,22], except in one more recent study [14] that focused on this species and found alpha-CoV and beta-CoV shed by bats. In the same way, alpha-CoVs were also identified in *Miniopterus schreibersii*, as described in other studies [15,16], but no beta-CoV was found, probably because of the small number of fecal samples tested (one in [15], two in [16], and five in our study).

The situation is more contrasted with *Pipistrellus pipistrellus*. Our results reinforce the findings of the other study carried out in France [2] with the identification of alpha-CoV and a prevalence of 2.44% (CI 95%: 1.05; 5.58) in our study versus 5.4% (CI 95%: 0.6; 7.9) in Goffard et al. [2]. However, *Pipistrellus pipistrellus* may be infected by beta-CoV as reported in Romania [1], while both beta- and alpha-CoVs were found in this species in the Netherlands [18], but also when considering the *Pipistrellus* genus in general [1,6,21].

This study demonstrated the circulation of beta- and alpha-CoVsin wild rabbits. Most of the detected nucleic sequences were very close to the beta-CoV of domestic rabbits in China [5], and the others were close to an alpha-CoV KU739072 detected from rodents in the UK [4]. Unfortunately, the lack of other studies targeting wild rabbits in its historical range prevents us from carrying out other comparisons. Continued surveillance of coronaviruses in rabbits would be useful to supplement our knowledge on infection in this species. Bearing in mind that wild rabbits are the ancestors of domestic rabbits, the study of coronaviruses in wild and domestic rabbits could be a useful model to better understand viral adaptive strategies. Indeed, human subtype OC43-related beta-CoV, Beta-CoV1 (lineage A), as identified is this study, is thought to have low host-specificity and to evolve by host-switching and recombination [5,14,23]. By contrast, alpha-CoVs are thought to be more specific to their hosts, at least in bats [21]. The identification in intestinal rabbit samples from twos department of alpha-CoVs very close to those identified previously (KU739072) in rodents [4] is not in favor of host specificity. Two hypotheses are possible. First, there is the circulation of non-specific alpha-CoVs within rodents, very close to viruses found in rabbits. The proximity of the *Rodentia* order to the *Lagomorpha* order, together forming the *Glires* clade [24], corroborates this hypothesis since rodents and rabbits share the same environment. Second, our sequences were short and were not sufficiently discriminant.

All sequences from rodents in this study were identified as beta-CoV, whereas in the study in the UK and Poland [4], all identified coronaviruses from rodents belonged to alpha-CoVs. Species that tested CoV-positive in the 2016 study were *Apodemus sylvaticus*, *Myodes glareolus*, *Microtus agrestis*, and *Rattus norvegicus*. The CoV-positive species in our study were *Myodes glareolus* and *Apodemus flavicollis*. No *Microtus* sp. tested were positive, and no *Rattus norvegicus* were sampled. As for bats and rabbits, the circulation of both coronaviruses should be expected in rodents in Europe, but further investigations are needed to confirm this hypothesis.

In addition, genetic segregation between coronaviruses from *Apodemus* and from *Myodes* was observed and suggested co-evolution with the host family [25]. The identification of one *Apodemus* infected by a coronavirus very close to that observed in *Myodes* may indicate potential host switching.

The situation seemed to be very different for hedgehogs with the observation of only beta-CoV. The prevalence in hedgehogs was higher than in other species. The only other study to our knowledge concerning coronaviruses in hedgehogs in Europe [3] found results for Germany that are very similar to those in our study, with a beta-CoV close to those that identified in this study (96% to 98% identity), and with a prevalence in fecal samples of 58.9%. By contrast, in the same animal shelter, two groups of coronaviruses separated by a 3.1% to 3.4% nucleotide distance in an 816-nt RdRp fragment were found, but all viruses were in the same clade. In our study, our short nucleic sequences of the RdRp gene were highly homogeneous, despite the significant distance between the three animal shelters in France. Hedgehogs are territorial; they do not travel distances as long as those between the shelters and they do not migrate. The similarity between sequences could be the result of co-evolution between viruses and hedgehogs.

This preliminary investigation of the presence of coronavirus in different wildlife species in France highlighted the presence of both alpha- and beta-CoVs in rodents, rabbits, and bats. The circulation of a range of coronaviruses within the same order or the same genus of mammals suggests the emergence of new coronaviruses by host switching, which is one of the main evolutionary mechanisms for coronaviruses [25].

In addition, our study highlighted observations in French wildlife compatible with the second evolutionary mechanism for coronaviruses reported by Anthony et al. [25]: Co-speciation, like for hedgehogs, *Apodemus* spp. and *Myodes glareolus*.

Our results showed the existence of potentially evolving virus strains, with possible crossing of the species barrier. Further investigations are needed in these species and other species of wildlife to assess the potential risk for domestic animals and humans of widely distributed CoVs by studying the spike region involved in the crossing of the species barrier [26,27].

## Figures and Tables

**Table 1:** Species of analyzed bats by year and by positive sample.

Species	Number of Carcasses Total (Positive Samples)			
	2013	2014	2015	total
Non-identified bats	2	4	3	9
*Rhinolophus ferrumequinum*	1	0	1	2
*Rhinolophus hipposideros*	4	2	2	8
*Rhinolophus euryale*	0	0	3	3
*Barbastella barbatellus*	1	3	0	4
*Myotis myotis*	0	1	0	1
*Myotis blythii*	0	0	1	1
*Myotis mystacinus*	0	1	4	5
*Myotis emarginatus*	1	11 (2)	1	13
*Myotis bechsteinii*	3	0	0	3
*Myotis daubentonii*	1	1	0	2
*Myotis nattereri*	0	0	1 (1)	1
*Myotis* sp.	0	0	1	1
*Nyctalus noctula*	2	0	0	2
*Nyctalus leisleri*	2	9	4	15
*Plecotus austriacus*	4	4	2	10
*Plecotus auritus*	3	4	8	15
*Pipistrellus pipistrellus*	42	127 (4)	78 (1)	247
*Pipistrellus kuhlii*	9	20	11	40
*Pipistrellus nathusii*	2	20	5	27
*Pipistrellus pygmaeus*	17	3	3	23
*Pipistrellus* sp.	11	20 (2)	7	38
*Vespertilio murinus*	0	1	1	2
*Eptesicus serotinus*	7	11	8	26
*Eptesicus nilssonii*	1	0	0	1
*Miniopterus schreibersii*	0	1 (1)	4 (1)	5
**Total**	113	243 (9)	148 (3)	504 (12)

**Table 2:** Number of samples and positive results for rodents per year and per area (NC: No capture program, NE: Not evaluable, [IC 95%]).

Year	Species	Bitche Infected/Total	Forbach Infected/Total	La Petite Pierre Infected/Total	Murbach Infected/Total	Wissembourg Infected/Total	Prevalence by Species
2014	*Apodemus* sp.	NC	6/82	0/4	0/0	0/0	(6/86) 6.98 [3.24; 14.40]
	*Myodes glareolus*	NC	3/32	0/4	0/2	0/0	(3/38) 7.89 [2.72; 20.80]
	*Arvicola terrestris*	NC	0/0	0/0	0/0	0/16	(0/16) 0 [0.00; 19.36]
	*Microtus* sp.	NC	0/0	0/0	0/0	0/3	(0/3) 0 [0.00; 56.15]
	Total	NC	9/114	0/8	0/2	0/19	
2015	*Apodemus* sp.	8/64	NC	1/38	0/2	0/0	(9/104) 8.65 [4.62; 15.63]
	*Myodes glareolus*	1/7	NC	0/17	1/15	0/0	(2/39) 5.13 [1.42; 16.89]
	*Arvicola terrestris*	0/0	NC	0/0	0/0	0/18	(0/18) 0 [0.00; 17.59]
	*Microtus* sp.	0/6	NC	0/0	0/0	0/0	(0/6) 0 [0.00; 39.30]
	Total	9/77	NC	1/55	1/17	0/18	
2016	*Apodemus* sp.	1/4	NC	0/11	0/1	NC	(1/16) 6.25 [1.11; 28.33]
	*Myodes glareolus*	0/2	NC	0/0	0/1	NC	(0/3) 0 [0.00; 56.15]
	*Arvicola terrestris*	0/0	NC	0/1	0/0	NC	(0/1) 0 [0.00; 79.35]
	*Microtus* sp.	0/0	NC	0/0	0/0	NC	NE
	Total	1/6	NC	0/12	0/2	NC	
**Total from 2014 to 2016**	*Apodemus* sp.	9/68	6/82	1/53	0/3	0/0	(16/206) 7.77 [4.84; 12.24]
	*Myodes glareolus*	1/9	3/32	0/21	1/18	0/0	(5/80) 6.25 [2.70; 13.81]
	*Arvicola terrestris*	0/0	0/0	0/1	0/0	0/34	(0/35) 0 [0.00; 9.89]
	*Microtus* sp.	0/6	0/0	0/0	0/0	0/3	(0/9) 0 [0.00; 29.91]
	**Total**	**10/83**	**9/114**	**1/75**	**1/21**	**0/37**	
